# Endogenous γ-Secretase Is Linked to Phagocytic Activity in Microglial Cells

**DOI:** 10.3390/s25113298

**Published:** 2025-05-24

**Authors:** Emily Williams, Mei C. Q. Houser, Sebastian Torres, Natalia Wieckiewicz, Michael Sadek, Midori Yokomizo, Masato Maesako

**Affiliations:** MassGeneral Institute for Neurodegenerative Disease, Massachusetts General Hospital, Harvard Medical School, 114, 16th Street, Charlestown, MA 02129, USA

**Keywords:** γ-secretase, FRET biosensor, microglia, phagocytosis

## Abstract

γ-Secretase has primarily been studied in neurons, whereas increasing evidence highlights its importance in microglia. Previous research has shown that the pharmacological inhibition of γ-secretase impairs microglial phagocytic activity. In this study, we used a genetically encoded Förster resonance energy transfer (FRET)-based biosensor to record γ-secretase activity, aiming to determine if naturally occurring cell-by-cell variations in endogenous γ-secretase activity are associated with phagocytic activity. Using the Notch1 N100 Y-T biosensor, we found that the regulation of endogenous γ-secretase activity varies among individual BV-2 microglial cells. Our multiplexed time-lapse imaging revealed that the phagocytosis of *E. coli* bioparticles was impaired in cells with lower γ-secretase activity compared to those with higher activity. Complementary biochemical analysis, utilizing Zymosan bioparticles and fluorescence-activated cell sorting (FACS), further demonstrated that cells with reduced phagocytic activity exhibited decreased endogenous γ-secretase activity. Collectively, our confirmatory study supports previous findings that microglial phagocytic activity is closely linked to γ-secretase and emphasizes the essential role of γ-secretase in microglia.

## 1. Introduction

Microglia are the resident macrophages of the central nervous system (CNS), and their normal function, activation, and dysfunction are tightly associated with development, aging, and brain diseases [1,2,3]. Microglia secrete cytokines and neurotropic factors that play pivotal roles in many aspects of immune responses in the CNS. Furthermore, microglia are responsible for phagocytosis, which is crucial for the removal of microbes, dead cells, and protein aggregates that could be harmful to neurons and their functions.

γ-Secretase is an enzyme complex responsible for the proteolytic processing of type I transmembrane proteins. The significance of γ-secretase is well established in neurons, particularly in the context of Notch signaling [4,5,6] and the processing of the amyloid precursor protein (APP) [7,8,9]. However, the role of γ-secretase in other cell types within the CNS remains unclear. Several studies have highlighted the involvement of γ-secretase in microglial migration and cytokine release (reviewed in [10]), while a more recent study has identified a pivotal role for γ-secretase in transcriptional regulation within microglia [11]. As described above, one of the essential functions of microglia is to phagocytose materials such as dead cells, debris, pathogens, and protein aggregates [1,2,3]. Notably, it has been reported that phagocytosis is impaired by the treatment of γ-secretase inhibitor [12], suggesting that γ-secretase is associated with phagocytic activity in microglia.

Several γ-secretase activity assays, such as the cell-free in vitro activity assay [13] and the cell-based reporter assay [14], have been previously developed; however, shortcomings in these assays do not permit an investigation of the dynamics of γ-secretase activity over time in individual cells. To better understand how endogenous γ-secretase activity is spatially and temporally regulated in live/intact cells, we recently developed Förster resonance energy transfer (FRET)-based biosensors for recording endogenous γ-secretase activity [15,16]. These biosensors have enabled us to detect that some cells exhibit higher while the others display lower γ-secretase activity, highlighting cell-by-cell heterogeneity in the regulation of endogenous γ-secretase activity. This unique capability of our biosensors has provided a new opportunity to ensure the correlation between γ-secretase and microglial phagocytic activity in more physiological settings. In this study, we aimed to address whether naturally occurring differences in endogenous γ-secretase activity define microglial phagocytic activities in individual cells.

Here, we record endogenous γ-secretase activity in BV-2 microglial cells using the Notch1 N100 YPet-mTurquoise-GL (N100 Y-T) biosensor [15]. Multiplexed time-lapse live-cell imaging revealed that phagocytic activity is impaired in the cells where endogenous γ-secretase activity is downregulated. Furthermore, we validated this finding using a complementary FACS and Western blot analysis. This new report confirms the previously identified link between γ-secretase and microglia phagocytosis and provides insights into how changes in endogenous γ-secretase activity regulate essential biological event(s) in various cell types.

## 2. Materials and Methods

### 2.1. Plasmid DNA, Antibodies, and Reagents

The plasmid encoding the Notch1 N100 Y-T biosensor was developed in a previous study [15]. An anti-APP C-terminus antibody was purchased from BioLegend (San Diego, CA, USA), anti-β-actin was from MilliporeSigma (Burlington, MA, USA), anti-cleaved Notch1 (Val1744) and anti-GAPDH antibodies were from Cell Signaling Technology, Inc (Dover, MA, USA), and anti-HA antibody was from Abcam (Cambridge, UK). γ-Secretase inhibitors DAPT and L-685,458 were purchased from Abcam and DMSO was from MilliporeSigma. pHrodo™ Red *Escherichia coli* (*E. coli*) BioParticles™, pHrodo™ Green Zymosan BioParticles™, and LysoTracker™ Deep Red were from Thermo Fisher Scientific (Waltham, MA, USA).

### 2.2. Cell Culture and Transfection

BV-2 cells were cultured in Opti-MEM Reduced Serum Medium (Thermo Fisher Scientific) with 5% FBS (Atlanta Biologicals Inc., Flowery Branch, GA, USA). The cells were authenticated using STR profiling and monitored for mycoplasma contamination. Lipofectamine 3000 (Thermo Fisher Scientific) was used for transient transfection according to the manufacturer’s instructions.

### 2.3. Confocal Microscopy and FRET

The Olympus FV3000RS Confocal Laser Scanning Microscope (Tokyo, Japan) was used for fluorescence imaging. The scope is equipped with a CO_2_/heating unit (Tokai-Hit, Fujinomiya, Japan) to maintain a suitable CO_2_ concentration and heating for live-cell imaging. Furthermore, the scope is equipped with the TruFocus Z drift compensation module to maintain focus during time-lapse imaging. A 10x/0.40NA objective was used for image acquisition.

For FRET detection, the cells expressing the N100 Y-T biosensor were excited by a laser at 405 nm, and the emission was simultaneously detected within 460–490 nm and 520–540 nm. A 520–540 nm over 460–490 nm emission ratio (i.e., Y/T ratio) was used as a readout of FRET. Pseudo-colored FRET images were generated using MATLAB version 8.4 (MathWorks, Natick, MA, USA). pHrodo™ Red *E. coli* BioParticles™ was excited by a laser at 561 nm, and emission was detected within 580–660 nm. A laser at 640 nm was used to excite LysoTracker™ DeepRed, and emission was detected within 670–770 nm. ImageJ version 1.54f was used to measure fluorescent intensity in regions of interest (ROIs).

### 2.4. Fluorescence-Activated Cell Sorting (FACS) and Western Blot

The Bio-Rad S3e (Hercules, CA, USA) or BD FACSMelody™ cell sorters (Franklin Lakes, NJ, USA) were used for cell sorting. The cells incubated with pHrodo™ Green Zymosan BioParticles™ were excited by a 488 nm laser, and the fluorescence emission was detected within 510–540 nm.

Proteins were extracted from cells using RIPA buffer (MilliporeSigma) with the Halt Protease Inhibitor Cocktail (Thermo Fisher Scientific). The protein concentration was determined using the BCA Protein Assay kit (Thermo Fisher Scientific). The samples were mixed with NuPAGE^TM^ LDS Sample Buffer and NuPAGE^TM^ Sample Reducing Agent (Thermo Fisher Scientific) and boiled for 3 min. Then, the samples were subjected to SDS-PAGE on NuPAGE^TM^ 4–12% Bis-Tris Protein gels (Thermo Fisher Scientific), followed by transfer to nitrocellulose membranes (Thermo Fisher Scientific) using the iBlot™ 2 Gel Transfer Device (Thermo Fisher Scientific). The membranes were incubated with primary and corresponding fluorophore-conjugated secondary antibodies and developed using the LI-COR Odyssey CLx scanner digital imaging system (LI-COR Biosciences, Lincoln, NE, USA).

### 2.5. Statistical Analysis

GraphPad Prism 9 (GraphPad Software, La Jolla, CA, USA) and Excel were used for the statistical analysis. The D’Agostino and Pearson omnibus normality test was used to examine the Gaussian distribution of the data and the variance equality. The one-sample *t*-test, unpaired *t*-test, and Mann–Whitney U test were used to compare the data. The Pearson correlation coefficient was measured to determine if γ-secretase activity correlates with phagocytic activity and/or lysosomal pH. In graphs, the mean and standard deviation (SD) were used as the center value and error bar, respectively. Three independent experiments were, at least, performed to ensure the reproducibility of the results.

## 3. Results

### 3.1. Recording Endogenous γ-Secretase Activity in Individual BV-2 Microglial Cells Using the N100 Y-T Biosensor

**Figure 1:**
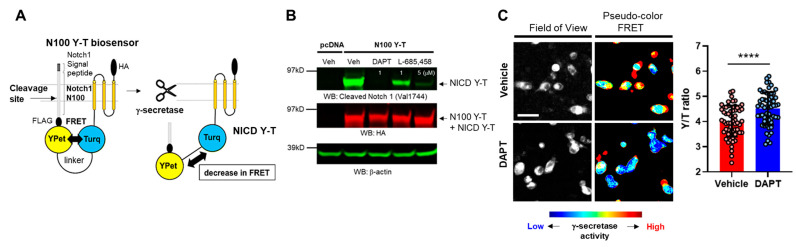
The N100 Y-T biosensor reports γ-secretase activity in BV-2 microglial cells: (**A**) schematic presentation of the N100 Y-T biosensor and its cleavage by γ-secretase. (**B**) Western blotting using HA and cleaved Notch1 antibodies. HA antibody captures both full-length N100 Y-T and γ-secretase cleavage product: Notch intracellular domain (NICD) Y-T. In contrast, the cleaved Notch1 antibody only recognizes NICD Y-T, showing its reduction by γ-secretase inhibitors: DAPT (1 µM), L-685,458 (1 or 5 µM) for 16 h of treatment. (**C**) Spectral FRET analysis shows that DAPT (1 µM) treatment significantly increases YPet/mTurquoise-GL emission ratio (Y/T ratio). Scale bar 20 µm, N = 60 cells, Mann–Whitney U test, **** *p* < 0.0001.

Pharmacological inhibition of γ-secretase is reported to impair phagocytic activity in microglial cells [12]. Here, we hypothesized that “naturally” altered endogenous γ-secretase activity is also associated with phagocytic activity in individual cells. To test the hypothesis, we employed the FRET-based Notch1 N100 Y-T biosensor, which enables recording endogenous γ-secretase activity on a cell-by-cell basis (Figure 1A) [15]. The N100 Y-T probe was transfected into BV-2 microglial cells, the cells were treated either with potent γ-secretase inhibitors DAPT (1 µM), L-685,458 (1 or 5 µM) or vehicle control for 16 hrs, and the cell lysates were subjected to Western blot. The Notch intracellular domain (NICD) Y-T fragment, which is produced from the N100 Y-T biosensor by γ-secretase cleavage, was significantly decreased by the treatment with γ-secretase inhibitors (Figure 1B), suggesting that endogenous γ-secretase successfully cleaves the N100 Y-T biosensor in BV-2 cells.

We then perform ratiometric spectral FRET analysis to determine if FRET between the donor and acceptor fluorophores within the N100 Y-T biosensor is changed due to γ-secretase cleavage. As such, BV-2 cells expressing the N100 Y-T biosensor were treated with 1 µM DAPT or vehicle control, the cells were excited by a laser at 405 nm, and emitted fluorescence from the donor (T: mTurquoise-GL) and acceptor (Y: YPet) was simultaneously detected using a confocal microscope. In image analysis, a region of interest (ROI) was generated over the entire cell, mTurquoise-GL and YPet fluorescence intensities were measured, and the average Y/T ratio was calculated for individual cells. We verified that the inhibition of the N100 Y-T biosensor cleavage by DAPT significantly increases FRET (Figure 1C), indicating that changes in FRET correlate with endogenous γ-secretase activity, and high FRET efficiency is associated with lower γ-secretase activity in BV-2 cells.

### 3.2. Phagocytosis Is Impaired in the BV-2 Cells with Lower γ-Secretase Activity

**Figure 2:**
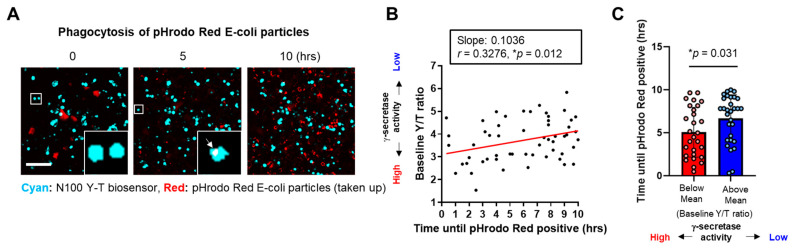
Time-lapse live-cell imaging reveals that BV-2 cells with lower γ-secretase activity display decreased phagocytic activity: (**A**) BV-2 cells expressing the N100 Y-T biosensor were incubated with pHrodo™ Red *E. coli* BioParticles™. γ-Secretase activity at the baseline was calculated, and the time until becoming positive with *E. coli* BioParticles™ was measured on a cell-by-cell basis. Scale bar 100 µm. (**B**) Positive correlation between baseline Y/T ratio (a higher ratio means lower γ-secretase activity) and the time until becoming *E. coli* BioParticles™-positive. N = 58 cells, Pearson correlation coefficient, * *p* < 0.05. R^2^ = 0.1073, RSME = 0.8619. MAE = 0.0002. (**C**) Significantly longer time until becoming pHrodo™ Red *E. coli* BioParticles™ in the BV-2 cells with lower γ-secretase (i.e., Y/T ratio is higher than the average mean) compared to those with higher activity. N = 28–30 cells, Mann–Whitney U test, * *p* < 0.05.

We next incubated BV-2 cells expressing the N100 Y-T biosensor with pHrodo™ Red *E. coli* BioParticles™ to perform multiplexed live-cell imaging analysis. These particles are pH-sensitive and thus non-fluorescent outside the cell but fluorescent bright red in phagosomes. In the imaging, we first conducted spectral FRET analysis to measure γ-secretase activity at the baseline on a cell-by-cell basis using the N100 Y-T biosensor, as described above. Then, we employed time-lapse live-cell imaging to track how long it took for individual cells to become positive for pHrodo™ Red *E. coli* BioParticles™ (Figure 2A). We discovered a statistically significant correlation between γ-secretase activity and the time to become *E. coli* BioParticles™-positive. Notably, cells exhibiting lower γ-secretase activity took longer to become *E. coli* BioParticles™-positive compared to those with higher activity (Figure 2B). We also divided the cells into two groups based on whether they were below or above the average mean of the Y/T FRET ratio at the baseline (t = 0), representing cell groups with higher or lower γ-secretase activity, respectively. We found that the cells with lower endogenous γ-secretase activity took a significantly longer time to become *E. coli* BioParticles™-positive compared to those with higher activity (Figure 2C).

**Figure 3:**
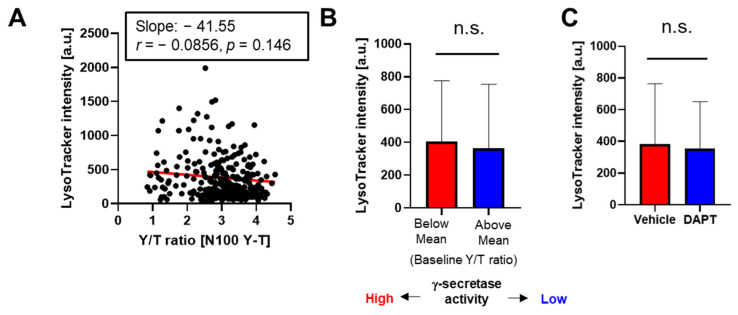
Not distinct lysosomal pH between the BV-2 cells with lower vs. higher γ-secretase activity: (**A**) BV-2 cells expressing the N100 Y-T biosensor were incubated with LysoTracker^TM^ DeepRed. γ-Secretase activity at the baseline was calculated and LysoTracker^TM^ DeepRed fluorescence was measured on a cell-by-cell basis, showing that there is no significant correlation. N = 289 cells. *p* = 0.146. Pearson correlation coefficient. (**B**) No significant difference in LysoTracker^TM^ DeepRed intensity between the BV-2 cells with lower γ-secretase (i.e., Y/T ratio is higher than the average mean) and those with higher activity (*p* = 0.07). N = 130–159 cells, Mann–Whitney U test. n.s. means not significant. (**C**) LysoTracker^TM^ DeepRed intensity is not significantly different between BV-2 cells treated with DAPT (1 µM) or vehicle control for 16 hrs (*p* = 0.91). N = 143–289 cells, Mann–Whitney U test. n.s.: not significant.

To eliminate the possibility that lysosomal pH rather than phagocytic activity is tightly associated with γ-secretase activity in BV-2 microglial cells, we incubated the cells expressing the N100 Y-T biosensor with LysoTracker™ Deep Red that records lysosomal pH. Unlike the pHrodo™ Red *E. coli* BioParticles™ (Figure 2), we found no correlation between LysoTracker™ Deep Red fluorescence and the Y/T FRET ratio representing γ-secretase activity (Figure 3A). When we divided the cells into groups based on their Y/T FRET ratio, LysoTracker™ Deep Red fluorescence showed no significant difference between the two groups (Figure 3B). Furthermore, we confirmed that LysoTracker™ Deep Red fluorescence did not differ between BV-2 cells treated with γ-secretase inhibitor DAPT or those with the vehicle control (Figure 3C).

**Figure 4:**
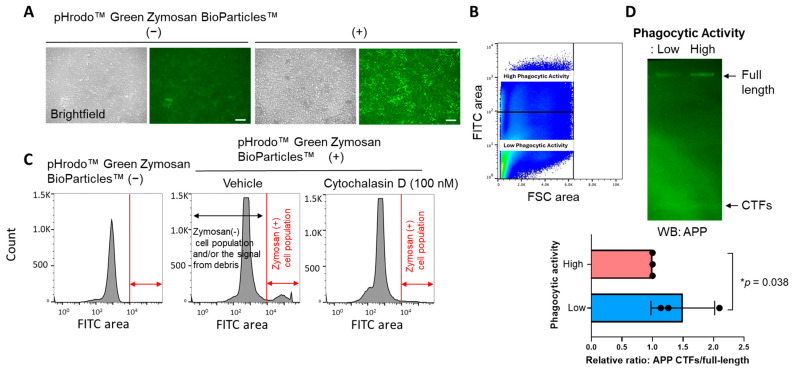
FACS and Western blot verify the link between γ-secretase and phagocytosis: (**A**) BV-2 cells phagocytosed pHrodo™ Green Zymosan BioParticles™, (**B**) which were subjected to FACS to isolate and extract the cells with higher and lower Zymosan BioParticles™ fluorescence. (**C**) The number of cells exhibiting Zymosan BioParticles™ positivity decreased by 100 nM cytochalasin D, a phagocytosis inhibitor, treatment. (**D**) The cell lysates from BV-2 cells with higher or lower Zymosan BioParticles™ fluorescence were subjected to Western blot using an APP C-terminus antibody. Increased APP-C-terminal fragments (CTFs), endogenous substrates of γ-secretase, over APP full-length ratio in the cells with lower phagocytic activity (1.497 ± 0.52 vs. high phagocytic activity set as 1) suggest decreased γ-secretase activity in the cell populations. N = 3 independent experiments. * *p* < 0.05, one-sample *t*-test.

Lastly, to verify the result from the time-lapse multiplexed imaging that utilizes *E. coli* BioParticles™, BV-2 cells were first incubated with pHrodo™ Green Zymosan BioParticles™ for 16 hrs (Figure 4A), and then the cells were subjected to FACS to be sorted out based on the Zymosan fluorescence (Figure 4B). The numbers of Zymosan-positive cells decreased by the treatment of cytochalasin D, a potent phagocytosis inhibitor [17,18], evidencing that the fluorescence is dependent on the phagocytosis of Zymosan BioParticles™ (Figure 4C). Then, the sorted cells with distinct phagocytic activities were subjected to Western blot using an APP C-terminus antibody to assess γ-secretase activity. Strikingly, we found that APP C-terminal fragments, immediate endogenous substrates of γ-secretase, over the APP full-length ratio are significantly increased in the cells with lower phagocytic activity compared to those with higher phagocytic activity (Figure 4D). This suggests that BV-2 cells with reduced phagocytic activity exhibit lower endogenous γ-secretase activity and further supports the link between γ-secretase and microglial phagocytosis.

## 4. Discussion

The great importance of γ-secretase and its pivotal roles in biology and diseases have been well established; still, how γ-secretase activity is spatial and temporally regulated remains unclear. Our recent development of genetically encoded FRET-based biosensors has allowed for endogenous γ-secretase activity to be recorded over time [15], on a cell-by-cell basis [15,16,19], with subcellular resolution [20,21], not only in vitro but also in vivo [22]. Moreover, the unique capabilities of the biosensors have enabled us to detect the cell-by-cell heterogeneity in endogenous γ-secretase activity and further determine the consequences of naturally altered γ-secretase activity in individual neurons (Yokomizo et al., 2024, in revision). In the present study, we employed the Notch1 N100 Y-T biosensor [15] to record γ-secretase activity in BV-2 microglial cells (Figure 1). Our findings indicate that phagocytic activity (Figure 2 and Figure 4), but not lysosomal pH (Figure 3), is decreased in cells exhibiting lower endogenous γ-secretase activity.

The membrane-bound enzymatic complex γ-secretase is responsible for the proteolytic cleavage of various transmembrane substrates [23]. The enzyme functions at a critical intersection between various cellular pathways such as Notch [4,5,6,24] and most notably APP proteolytic processing [7,8,9], where it facilitates the development of one of the pathological hallmarks of AD. γ-Secretase does not solely function in the APP pathway; thus, γ-secretase inhibitors alone are insufficient to slow down AD progression [25,26], despite demonstrating an ability to reduce the β-amyloid concentration in the CNS [27]. Several studies have highlighted the essential roles played by γ-secretase in microglia, including the modulation of pro-inflammatory cytokine expression and release [28,29] and the regulation of microglial migration [30].

The phagocytic clearance of cell debris, pathogens, and protein aggregates is one of the core functions of microglia [1,2,3,31]. The previous literature has connected γ-secretase to phagocytic activity using a γ-secretase inhibitor [12], establishing a clear cause-and-effect relationship. However, there is limited knowledge about whether microglial phagocytosis in individual cells relies on endogenous γ-secretase activity. Hence, we utilized our FRET-based biosensor and pHrodo™ *E. coli* BioParticles™ in time-lapse live-cell imaging and realized a heterogeneity of γ-secretase activity on a cell-by-cell basis, which correlated to a similar heterogeneity of microglial phagocytic activity (Figure 2). This correlation was further verified by pHrodo™ Zymosan BioParticles™ adapted to flow cytometry and cell sorting. We identified that the cells demonstrating reduced γ-secretase processing of APP-CTFs were also the cells that had a lower phagocytic efficiency of Zymosan BioParticles™ (Figure 4). Of note, we visualized a decrease in fluorescence in response to a phagocytic inhibitor cytochalasin D treatment, demonstrating the reliability of Zymosan fluorescence in capturing phagocytic activity (Figure 4). We also ensured that our measurements using pHrodo™ *E. coli* BioParticles™ truly represented phagocytosis rather than changes in lysosomal pH (Figure 3). Altogether, this study employed two different assays (i.e., time-lapse imaging, FACS cell sorting and Western blot) and two distinct bioparticles (i.e., pHrodo™ *E. coli* and Zymosan BioParticles™) to validate the correlation between phagocytosis and endogenous γ-secretase activity.

Microglial phagocytic activity is regulated by γ-secretase; however, the underlying molecular mechanisms are not fully understood. The triggering receptor expressed on myeloid cells 2 (TREM2) is a type I membrane protein exclusively expressed in microglia [32] and is tightly associated with microglial phagocytic activity [33,34,35]. TREM2 undergoes the regulated sequential intramembrane proteolysis first by α-secretase(s) and then by γ-secretase [36,37,38,39]. To initiate downstream signaling upon ligand activation, TREM2 requires a complex formation with adapter proteins (e.g., DAP12) [32]. It has been reported that the inhibition of γ-secretase activity decreases TREM2-dependent phagocytosis in BV-2 cells [40]. Mechanistically, γ-secretase inhibition results in the accumulation of TREM2 C-term fragments (TREM2 CTFs), limiting the interaction of DAP12 with the functional full-length receptor and thus decreasing DAP12 phosphorylation [36]. TREM2 may, therefore, link between diminished endogenous γ-secretase activity and microglial phagocytosis. Detailed mechanistic research is necessary to establish the exact molecular link between γ-secretase and microglia phagocytosis. Furthermore, phagocytic behavior and regulating factors in BV-2 cells may differ from those of primary microglia and/or microglia in the brain in vivo; thus, validation of the findings in more physiologically relevant models is also necessary.

Lastly, the Y/T ratio from the N100 Y-T biosensor is correlated with the time until pHrodo™ *E. coli* BioParticles™ positivity in a statistically significant manner (Figure 2C), and its Pearson correlation coefficient (*r* = 0.3276) is larger than the cut-off used in biological science [41,42]. However, the correlation is mild; therefore, the possible reasons behind the non-robust correlation would be worthwhile to be discussed. First, we think it could be because microglia phagocytic activity is regulated not only by γ-secretase but also by many other factors. Second, the non-robustness could be also related to the design of our biosensor. Whereas we are confident that our FRET biosensor can sensitively record endogenous γ-secretase activity over time on a cell-by-cell basis in live cells, which are fully validated by the series of our previous publications [15,16,19,20,21,22], the exact “purity” of the correlation between the Y/T ratio and γ-secretase activity remains unclear due to the logistics behind the design of the biosensor. The FRET phenomenon relies on orientation and proximity between the donor and acceptor fluorophores. To make our biosensor more proximity-dependent, excluding the contribution of orientation and aiming to increase sensitivity, we optimized the linker length between the donor and acceptor fluorophores from 20 amino acids to 80 amino acids [15]. Yet, our recent findings using multiple different cell types in vitro and in vivo suggest that orientation still plays a role [22]. These findings are particularly important since our FRET biosensor, the N100 Y-T biosensor in this study, uses the SAGG repeat flexible linker, which randomizes the orientation between the donor and acceptor of the sensor as well as that of the biosensor cleavage product. Therefore, we expect that this randomness may create the “dirtiness” in the Y/T ratio, contributing to the appearance of a less striking correlation between the Y/T ratio and the time until pHrodo™ *E. coli* BioParticles™ positivity.

## 5. Conclusions

This study verifies a previously discovered link between γ-secretase and phagocytosis in BV-2 microglial cells. Using the N100 Y-T biosensor, we demonstrate that 1) endogenous γ-secretase activity is differently regulated in individual BV-2 cells and, while the correlation is not strong, 2) phagocytic activity is reduced in the cells with diminished endogenous γ-secretase activity. This finding is consistent with earlier research that reported impaired phagocytosis following γ-secretase inhibitor treatment [12]. This study also sheds light on the capability of genetically encoded biosensors to record endogenous γ-secretase activity with single-cell resolution, facilitating a deeper understanding of the spatiotemporal regulation of γ-secretase and its consequences in various cell types beyond neurons.

## Data Availability

All data are visualized and included in the manuscript. The raw data used to create the corresponding graphs can be shared by the corresponding author upon reasonable requests.

## Abbreviations

The following abbreviations are used in this manuscript:

APP	amyloid precursor protein
FACS	fluorescence-activated cell sorting
FRET	Förster resonance energy transfer
Y-T	YPet-mTurquoise-GL
